# Family Medicine Residents’ Perspectives in Dermatology Training

**DOI:** 10.15694/mep.2021.000090.1

**Published:** 2021-04-08

**Authors:** Jessica Howard, Lauren Hayley Siegel, Kelly Mok, Shannon Sibbald

**Affiliations:** 1 Schulich School of Medicine & Dentistry

**Keywords:** medical education, dermatology, rural health services

## Abstract

This article was migrated. The article was marked as recommended.

**Purpose:**Dermatological concerns are one of the most common presentations in family practice. Residents often feel inadequately prepared to address these concerns after graduation. This preliminary qualitative study was conducted to gain insight from residents about their post-graduate training in dermatology with a family physician practicing in dermatology.

**Methods:**Family medicine residents (within five years of graduation) who completed their training at an academic family medicine centre in rural Southwestern Ontario affiliated with the local University’s Department of Family Medicine were interviewed (n=7). Phenomenological analysis was performed on the interviews using qualitative principles of immersion and emergence.

**Results:** Three main themes based on training with a family physician were identified: 1) Personal Enrichment, 2) Professional Enrichment, and 3) Understanding of System Barriers and Facilitators.

**Conclusion:** Training with a family physician practicing in dermatology is useful for residents to gain comfort and skills in the discipline, allowing them to better serve their patients when in independent practice.

## Introduction

The family physician’s office is commonly the first place where patients present with skin conditions, with up to one-quarter of primary care visits involving skin disorders ( Branch, Collins and Wintroub, 1983; Kerr *et al.,* 2010).The ability of family physicians to identify and treat common dermatologic conditions has been shown to reduce wait times for referrals to specialists and increase patient satisfaction ( Chuh *et al.*, 2005; Merenstein *et al.*, 2007). The shortage of practicing dermatologists in Canada has been well-documented ( Chow and Searles, 2010; Heughan *et al.,* 2008). In general, wait times for health services have increased over the last decade, with patients living in rural areas experiencing significantly longer wait times compared to their urban counterparts ( Sibley and Weiner, 2011; Yadav *et al*., 2016). According to a 2016 report ( Bosco and Oandasan, 2016), only 3.1% of specialists practice in rural areas in Canada.A 2018 study based in Ontario, Canada ( Micieli and Alhusayen, 2018) reported that there were 0.47 dermatologists per 100,000 people in rural areas compared to 1.96 dermatologists per 100,000 people in urban areas. There is a need for family physicians to be able to accurately diagnose and treat common dermatological problems, especially in rural communities where there are a lack of specialists.

The increasing incidence of skin conditions in an aging population have contributed to the increased demand for dermatological procedures ( Dall *et al.,* 2013; Kosmadaki and Gilchrest, 2013). With proper training, family physicians are capable of performing diagnostic and treatment procedures in dermatology with good correlation to dermatologists ( Chuh *et al.*, 2005; Merenstein *et al.*, 2007). A study by Wetmore *et al.* (2005) presented a comprehensive list of procedures that a graduate from a Canadian family practice training program should learn and be able to perform ( Table 1). However, the time in which trainees are given to acquire these skills is limited. As such, efforts have been taken to increase the amount of education medical students receive in dermatology. A 2017 survey of medical schools across Canada ( Hu and Vender, 2018) revealed that an average of 25.6 hours of undergraduate education was dedicated to dermatology, a 25% increase in teaching time compared to 2008. Despite an increase in the average number of faculty members specializing in dermatology between 2008 and 2017, a lack of faculty was frequently cited as a barrier to allocating more teaching time to dermatology ( Hu and Vender, 2018).

**Table 1.** Skin Procedures

Procedures	
Incise and drain abscesses	Insert sutures
Repair laceration	Perform skin biopsies
Excise dermal lesions	Remove a foreign body
Perform wound debridement	Perform cryotherapy
Perform electrocautery	Scrape skin for fungus
Use a wood lamp	Release subungual hematoma
Drain acute paronychia	Partially remove a toenail
Perform wedge excision	Pare skin callus

(adapted from Wetmore *et al.,* 2005)

There is a lack of empirical evidence on family physicians’ views towards undergraduate and postgraduate training in dermatology regarding the effectiveness of training and usefulness of these skills in practice. We set out to fill this gap in the literature through a qualitative study of recently graduated family medicine residents. The purpose of this qualitative study was to gain a first-hand account of post-graduate training in dermatology from residents that completed their Family Medicine Residency block at a family medicine clinic, where they received extra dermatology-specific training. It was expected that the results of this preliminary study would provide novel insight into post-graduate medical training with the specific intention of guiding quality improvement initiatives in the organization of family medicine residency programs.

## Methods

This study employed a qualitative, phenomenological approach to data collection and analysis ( Dowling, 2007). Qualitative methodologies are increasingly popular in the field of health care because they allow for the unbiased collection and presentation of participant experiences ( Meyer, 2000). While novel findings may not be generalizable to other settings, lessons may emerge which can be used to restructure and enhance existing practice. This study was approved by the Health Sciences Research Ethics Board at Western University (Protocol #109008).

### Setting

Middlesex Centre Family Medicine Clinic (MCFMC), in rural Southwestern Ontario, is an academic clinic affiliated with the Department of Family Medicine at the Schulich School of Medicine and Dentistry, Western University.

The MCFMC is a core teaching site for the regional postgraduate family medicine program at Western University, situated in Ilderton, Ontario and is staffed by six family physicians. The patients of MCFMC are primarily those who live in the rural setting and are employed in agricultural professions.

A unique educational opportunity for residents at MCFMC is the participation in ‘skin clinics’. Skin clinics are run once a week by one of the family physicians at the centre (author JH) who holds a diploma in Practical Dermatology and a focused practice designation in dermatology. This allows residents to receive additional, hands-on training in dermatology, which is not available to the majority of residents that complete their training at other Canadian family medicine clinics.

### Participants and recruitment

Residents that completed their Family Medicine Residency Block at MCFMC within five years prior to the time of this study were recruited to participate in this study (n= 7). Participants were recruited by a secondary investigator (LHS) via email and provided with a letter of information and consent. Interviews continued to be scheduled until saturation was reached (when no new concepts were discovered during analysis of interviews). No compensation was provided to participants.

### Interviews

**Figure 1.**
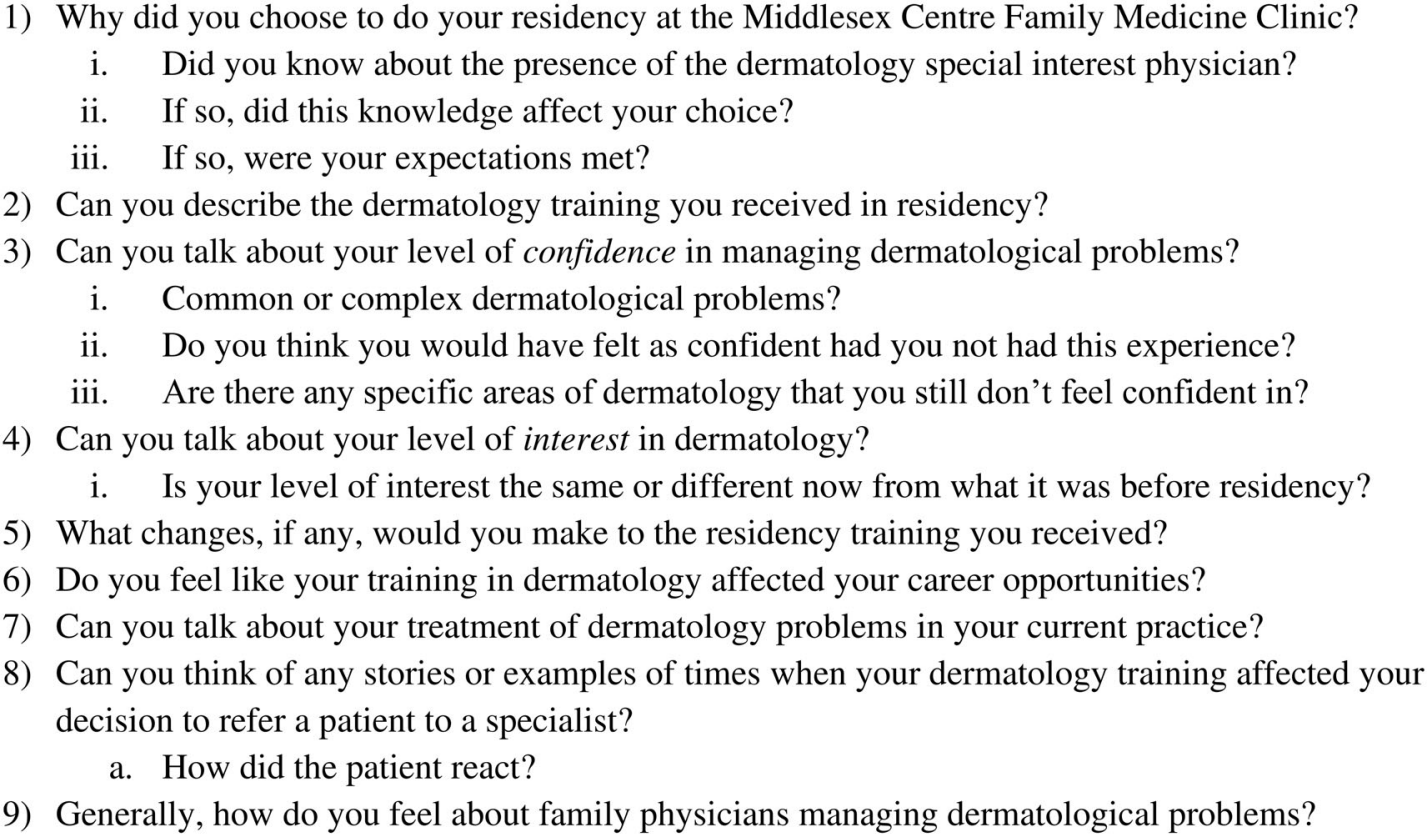
Semi Structured Interview Guide.

A semi-structured interview guide was developed after a review of the literature, namely papers by Uzuner *et al.* (2010) and Bosco and Oandasan (2016), followed by discussion and consensus from all members of the research team. Each participant participated in a 1:1, semi-structured interview, conducted by a secondary investigator that had no prior relationship with or authority over the participants. Following phenomenological guidelines, the interviewer allowed the participants to guide the interview to the topics that they found relevant or skip questions that they did not find relevant ( Smith and Osborn, 2003). In keeping with the iterative nature of qualitative methods, the interview guide underwent modification during the data collection process as emerging themes led to the development of new questions and the removal of others. Interviews were guided by nine open-ended questions and related probing statements ( Figure 1).

### Interview analysis

Audio recordings were transcribed verbatim by a transcriptionist and all identifiers were removed. Transcripts were then coded and sorted into content areas with the support of on-line analysis software (Dedoose). Each of the content areas were read in detail and summarized into emerging patterns using the qualitative principles of immersion and emergence ( Thorne, 2000). New data were compared to emerging themes from previous transcripts in an iterative and reciprocal manner ( Barnett-Page and Thomas, 2009). Field notes were reviewed and incorporated into the analysis to verify and validate findings. Themes and sub-themes were developed to reflect the most salient patterns within and across coded topic areas. This study also employed the ‘constant comparison’ method to make comparisons at each stage of the analysis to identify similarities and differences within and across interviews ( Miles and Huberman, 1994).

In order to ensure credibility and validity of the analysis, a multidimensional research team consisting of individuals with varying experiences in the analysis of the data; this supported triangulation of our findings ( Barnett-Page and Thomas, 2009). Triangulation improves validity of the results by allowing multiple perspectives to explore issues, despite the inherently different biases and strengths of those involved ( Barnett-Page and Thomas, 2009). Emerging interpretations were reviewed and challenged by the research team and discrepancies were resolved via discussion and consensus of the team ( Charmaz, 2006).

## Results/Analysis

Seven residents that completed their Family Medicine Residency Block at MCFMC within five years prior to the time this study began were recruited to participate in this study ( Table 2). In total there was one male and six females. Two participants were in residency at the time of the study and the remaining five ranged from one to four years since completing their residency. Two participants were directly supervised by the special interest dermatology physician (JH) and the remaining five were supervised by other physicians at the clinic. All residents at the centre received more exposure to dermatology than residents at other Ontario centres.

**Table 2.** Demographic information of participants

Participant ID	Sex	Supervisor (JH or Other)	Years since residency
LY	M	Other	1
AO	F	Other	4
YH	F	JH	1
JS	F	Other	2
DZ	F	JH	1
KR	F	Other	0
HY	F	Other	0

From data analysis, three main themes were identified: 1) *Clinical Competency* 2) *Transferrable Skills*, and 3) *Health System Challenges* ( Table 3) *.* Quotes that illustrate each theme have been provided in Table 4.

**Table 3.** Themes describing the experience of dermatology training in residency

Theme	Sub-themes
Clinical Competency	Confidence
	Comfort
	Satisfaction
Transferrable Skills	Diagnosis and treatment
	Transferable skills
	Patient-centered care
Health System Challenges	Referrals
	Gap in Canadian healthcare
	Urban/rural care

**Table 4.** Quotations describing the experience of dermatology training in residency

Theme	Quotation Number	Quotation
Clinical Competency	1	*“I really can’t stress enough the importance of seeing a volume of skin lesions and presentations because it really is something that, you know, takes a trained eye and, and you just cannot get that out of a textbook or out of images.... So, I do feel way more confident identifying some of the more obscure and more sinister presentation that you can see in dermatology.” (LY)*
Clinical Competency	2	*“I think I was always good with my hands, but now.... I have more of a comfort of what I can and cannot handle.” (HY)*
Clinical Competency	3	*“I’m happy to take five minutes and address something because it’s easy for me and it be a benefit for the patient.” (LY)*
Clinical Competency	4	*“It, it feels nice to, to, to be able to look at something and know what, what it is or think you know what it is; and at least have a couple of different ideas, if you don’t know of what it could be and the way you can attack the problem, and things you can try before you have to refer.” (KR)*
Transferable Skills	5	*“I’m much more comfortable with that, having had, had the experience in [place]. And then the initial management, like you know your first top three questions for certain things. I’m willing to, you know, pursue those before I would probably just say ‘I don’t know what this is’, or ‘I don’t really know what to do about it.’” (KR)*
Transferable Skills	6	*“I found that I was actually in my electives when I was kind of the most expert person on the internal medicine team about how to deal with a vasculitis type rash; and how to appropriately biopsy it; what mediums to send it in,; and what particularly you were sending it for but that’s where I really felt like all my training has equipped me to deal with real-life problems in a special way.” (DZ)*
Transferable Skills	7	*“...I’m back on electives and I’m back in the hospital. And so, you know, suturing a central line or even just, you know, doing that skin punch biopsy to rule out vasculitis on internal medicine, whereas some of the internal medicine residents haven’t had all of that same hands-on skin training has left me feeling, you know, like I’m competent and confident in doing those with, you know, no supervision.” (DZ)*
Transferable Skills	8	*“I can provide a couple of stories where even in my early clinical practice; I’ve been able to pick up sinister lesions pretty quickly. And even, even in the emergency room, you know, identifying sinister rashes that are, are not just limited to a dermatological disease is, is an important skill set.”* (LY)
Transferable Skills	9	*“...as a junior resident, I remember we had a young girl who was probably 13, just about to go for Grade 8 grad the following month. And she had pretty bad acne and not, not Accutane worthy quite yet but definitely pretty severe acne. And we had probably another two or three months to go before her grad so we had some time to make a, make a, make a difference with her acne. And it was just, it was so lovely being able to tell her that we could make it quite a bit better before her grad. And just the way her face lit up, it was just, it was just lovely. It makes you feel good about what you’re doing.” (HY)*
Transferable Skills	10	“ *I think most of the patients are very happy when their family doctor does additional procedures or additional work-up rather than feeling anxious and waiting for referrals. You know, there’s a small subset of, of patients who prefer to see specialists, but I think the vast majority of people want their problem dealt with as quickly and efficiently as possible.” (DZ)*
Health Systems Challenges	11	*“I know some of these common things and can save my patients... like I can appropriately treat them instead of having them worry and wait for, you know, six months to a year to see a dermatologist.”* (JS)
Health Systems Challenges	12	*“I do some things different than my colleagues at the clinic I’m at now. So, for example, like immunofluorescence. Like they would never send, they never had the bottles. They never really checked that off...So that way, that’s an extra step for patients, like that’s a step for patients that’s at least is getting done and not, you don’t have to wait for dermatology then to do that.” (AO)*
Health Systems Challenges	13	*“I definitely feel that I still refer to Dermatology especially for things that are beyond my level of training and my comfort level. So a lot of times if I’m concerned about patients that have very extensive disease for whatever, whether it be psoriasis or acne and things like that, sometimes I do get a second opinion just to make sure that before starting very aggressive therapy such as oral retinoids or even a biologic, that I do confirm that the diagnosis is correct, and that there aren’t any other treatment options that I haven’t exhausted.” (YH)*
Health Systems Challenges	14	*“As family docs . . . skin is a huge part of {sic} the patients coming through the door. And if you’re not equipped, then we just burden, like there is lots of stuff we should be able to treat confidently that we’re referring to tertiary dermatologists, which they’re way over-qualified to treat.”* (JS).
Health Systems Challenges	15	*“I think it is a good use of resources. I think from a system point of view there’s no need for simple, basic, you know, common conditions to need to be managed with an expensive consulting code . . . You can see here in [place], much difference with GP dermatology like because there’s this little pocket here and Dr. [name] and some other clinics” (JS)*
Health Systems Challenges	16	*“I think for the patient population, rurally, it’s more important because you don’t have access. So, knowing what to do or knowing how to do it is important”* (YH)
Health Systems Challenges	17	*“...Certainly, in rural/regional areas, patient access just to coming to a town is much more difficult. It’s usually a further drive and they just don’t have the means to get into a larger centre. So, if their GP can provide that service, I think it is fantastic. So, I would say probably more needed in a rural community and better to have those additional skills if you are more of a rural/regional physician.”* (KR)

### Theme 1: Clinical Competency

The theme ‘ *Clinical Competency’* includes three subthemes; confidence, comfort and satisfaction. Residents felt that their confidence in dermatology improved as a direct result of the increased training they received in dermatological procedures, whether JH was their immediate supervisor or simply available for consultation at the clinic. LY explained that her confidence was improved simply in terms of the volume of patients and procedures to which she was exposed. Another participant explained that he was more comfortable with dermatology as a result of the training. In addition, participants expressed improved personal satisfaction with their competence and abilities to treat dermatological problems.

### Theme 2: Transferrable Skills

Diagnosis and treatment of dermatological problems is a complex process. Participants felt that their experiences in the clinic not only enabled them to enhance their skills, but also had them challenging themselves to diagnose before second-guessing *.* From a professional standpoint, the majority of participants felt that the additional training they received in dermatology made their skills transferrable to other areas of medicine. One participant felt that what they learned in clinic helped build their confidence in being able to do procedures outside of their family medicine rotations and another participant explained applying his dermatology training in the emergency room. Lastly, participants revealed the ability to provide better patient-centered care as a result of their dermatology training.

### Theme 3: Health System Challenges

The third theme, ‘ *Health System Challenges’*, contains the sub-themes referrals, gap in Canadian healthcare, and urban/rural care. Many participants spoke of the benefit of being able to treat dermatological problems as a family physician without having to refer to a dermatologist. AO described a specific task that she was able to do that family physicians without dermatology training might not. However, many participants also expressed that they knew their limits and would still refer to a specialist as needed.Addressing gaps in the Canadian health care was a subtheme that was articulated by several participants. Many participants thought that family physicians were filling a need in the health care system by addressing dermatological problems. This was thought to be taking away some of the burden from an already taxed system. When asked how they felt about family physicians managing dermatology, a resident responded by saying that they believed it to be a good usage of resources. Of importance to some residents was how dermatological training by family physicians was addressing a need in the rural communities, given the difficulties it may cause for some patients to commute.

## Discussion

Family physicians are well-positioned to treat and manage skin problems, especially in our over-burdened healthcare system ( Chuh *et al.*, 2005; Merenstein *et al.*, 2007). The presence of a teaching centre in Southwestern Ontario with a family physician who has a focused practice in dermatology has created a unique opportunity for family medicine residents. At this centre, residents receive additional hands-on training in dermatology and the opportunity to participate in weekly “skin clinics”. Due to the novelty of this experience, we believed gaining a first-hand account of resident experiences from the residents involved was important to better understand perceived benefits for future research and allow potential lessons to be shared more broadly.

This qualitative study revealed many positive outcomes overall. Participating residents described improvements not only in their skills in dermatology, but also in their comfort and confidence with these skills. Participants described their decreased need to refer to specialists and the subsequent benefit to patients and the overall healthcare system. Ultimately, training in this unique environment led participants to describe increased personal satisfaction and better patient-centered care.

There are several recommendations to improve practice across family medical clinics, and medical education more broadly. Firstly, the quality of dermatology training at the undergraduate levels should be improved. This should include didactic teaching as well as more opportunities for hands-on learning. Secondly, residents would benefit from increased training opportunities at the postgraduate level and continued professional development beyond this. Although dermatology teachings in Canadian medical schools has increased between 2008 and 2017, the overwhelming majority of residents are uncomfortable assessing and managing dermatological issues in patients. Providing increased clinical rotations with participation from community dermatologists and teaching of clinical skill sessions can provide a higher level of clinical exposure to residents for increased learning opportunities.Lastly, teaching centres would benefit from the increased presence of teaching physicians with speciality training.

### Limitations

In part because of the small population from which to recruit from and also because of our target of residents training with family physicians practicing dermatology, we completed this study with a small sample. Although this study did reach saturation, a larger sample would have allowed a broader perspective into dermatological training in family medicine residency. Furthermore, all participants completed their family medicine residency at MCFMC, which may have led to a uniform demographic. However, clinical observations outside of the study corroborate the responses given by the participants. Future studies may benefit from conducting qualitative analyses of both residents and family physicians that completed their residency at different clinics in both rural and urban settings.

## Conclusion

Dermatological diagnostic and procedural skills remain a perceived deficit by family medicine residents and physicians. This is supported by evidence for more dermatological training in medical school and residency to improve comfort with these skills to better serve patients. The intriguing initial observations from our study demonstrate that training with a family physician practicing in dermatology can elevate competency and skills in dermatological care as well as more broadly.

## Take Home Messages

• Primary care physicians with training in dermatology can help to address gaps in the healthcare system
• Dermatological skills remain a perceived deficit by family medicine residents; there is need for more didactic teaching and hands-on learning in dermatology during residency
• Clinical rotations with community dermatologists can help residents improve their dermatological skills and ability to provide patient-centred care

## Notes On Contributors

**Jessica Howard**, MD, CCFP, Dip P Derm, is an Assistant Professor in the Department of Family Medicine at the Schulich School of Medicine & Dentistry, Western University in London, Ontario. Dr. Howard is a practicing family physician who holds a diploma in Practical Dermatology and a focused practice designation in dermatology.

**Lauren Hayley Siegel**, MSc, is the clinical faculty research coordinator for the Department of Family Medicine at the Schulich School of Medicine & Dentistry, Western University in London, Ontario.

**Kelly Mok** is an undergraduate student in the Department of Epidemiology & Biostatistics at Western University in London, Ontario.

**Shannon Sibbald**, PhD, MSc, is an Assistant Professor in the School of Health Studies, Department of Family Medicine, and Schulich Interfaculty Program in Public Health at Western University in London, Ontario. Dr. Sibbald’s interests and field of research are within interdisciplinary health and health systems research as well as implementation science ORCID ID: https://orcid.org/0000-0002-4328-6489
