# Supplementary material for: Family Medicine Residents’ Perspectives in Dermatology Training
Source: MedEdPublish (2016). 2021 Apr 8;10:90. [Version 1] doi: 10.15694/mep.2021.000090.1 (PMC10939655; doi:10.15694/mep.2021.000090.1)
Supplement: Peer review history

# Reviewer report: Reviewer response for version 1

**DOI:** 10.21956/mep.18864.r26907

**Published:** 2021-06-18

**Authors:** Ken Masters

**Affiliations:** 1 Sultan Qaboos University

This review has been migrated. The reviewer awarded 3 stars out of 5 An interesting qualitative study dealing with family medicine residents’ perspectives in dermatology training. The Introduction gives a good background on dermatology training needs and offerings in Canada, and then points to the need as expressed by family physicians regarding suitable training.Although the study is small and somewhat localised, it does offer valuable insight into the topic.My main reservations, however, are with the labelling of the Methodology, and I would like the authors to re-evaluate this:• The authors say that they have conducted a phenomenological study, and then give Dowling 2007 as their source. This is most unfortunate, as Dowling gives an overview of the wide range of phenomenological approaches, even up to the modern split between “American” and “European” phenomenology; one area that many regard as crucial to a phenomenological study is the concept of bracketing, but the researchers of this paper make no mention of bracketing in the Methods. So, the fault here is that the researchers state that they are following a methodology based on a theoretical approach, but their source does not directly reflect that single theoretical approach. To rectify this, the authors should rathero indicate _which_ phenomenological approach in Dowling is being used, oro select a source that allows them to more accurately ground their Methodology in the theoretical approach, oro if they are using an eclectic combination of different phenomenological approaches, then identify the elements used, oro not position their Methodology in the theoretical approach at all.• In fact (although more details would be required), from the brief description of the interviews and simultaneously theming and then stopping at saturation, it appears that the researchers may have drawn heavily on Grounded Theory. But more details would be required to establish that (and some of the top-level questions are more specific than one would expect in Grounded Theory, but these are outweighed by the larger number of open-ended top-level questions).• “Qualitative methodologies are increasingly popular in the field of health care because they allow for the unbiased collection and presentation of participant experiences” The researchers cite Meyer 2000. Again, it appears that the researchers are on shaky ground. Julienne Meyer speaks about “Action research”, and, although the author says that action research frequently draws on qualitative methods, makes it clear that action research is “not synonymous with qualitative research”. Nowhere in that paper do I see the point that “Qualitative methodologies…allow for the unbiased collection and presentation of participant experiences.” It would be useful for the researchers to address this anomaly.So, I think the study is necessary, and has been well-conducted and presented, but the labelling and theoretical descriptions of the Methods need to be reviewed and clarified. I look forward to Version 2 of the paper in which these issues are addressed.

Reviewer Expertise:

NA

No decision status is available

# Reviewer report: Reviewer response for version 1

**DOI:** 10.21956/mep.18864.r26906

**Published:** 2021-04-09

**Authors:** sathyanarayanan varadarajan

**Affiliations:** 1 SRM Medical College, SRM University

This review has been migrated. The reviewer awarded 4 stars out of 5 This is an interesting qualitative study done with the purpose of gaining insight from residents about their post-graduate training in dermatology with a family physician practicing in dermatology. Recruited seven participants were interviewed 1:1. The Interview was semi-structured and were guided by nine open-ended questions and related probing statements. Phenomenological analysis were performed on the interviews using qualitative principles of immersion and emergence by a multidimensional research team consisting of individuals with varying experiences in the analysis of the data. From data analysis, the authors identified and discussed the results with three themes (Clinical Competency, Transferable Skills, and Health System Challenges). The authors provide several recommendations to improve practice across family medical clinics, and medical education broadly through their observation. They conclude that training with a family physician practicing in dermatology can elevate competency and skills in dermatological care as well as more broadly.Though this valid and credible qualitative study has few limitations such as small sample size, it contributes valuable insights to improve practice and medical education.

Reviewer Expertise:

NA

No decision status is available
